# Magnetic Beads for Schistosomiasis Diagnosis

**DOI:** 10.1371/journal.pntd.0000159

**Published:** 2007-12-26

**Authors:** Malcolm K. Jones, Julie Balen

**Affiliations:** 1 Australian Centre for International and Tropical Health, Queensland Institute of Medical Research, Herston, Queensland, Australia; 2 School of Veterinary Science, The University of Queensland, St Lucia, Queensland, Australia; 3 School of Population Health, The University of Queensland, St Lucia, Queensland, Australia; George Washington University, United States of America

## Background

Schistosomiasis is a chronic helminth disease of humans, linked persistently with poverty. Parasite transmission is reliant on poor sanitation for transmission from humans to the molluscan intermediate host [1],[2]. Recent estimates of the global disease burden for schistosomiasis indicate that some 207 million people are infected, with over 750 million people at risk [2]. New control strategies include evidence-based approaches, which incorporate targeted chemotherapy in areas of low endemicity (≤10% prevalence) and mass drug administration in regions of moderate to high endemicity [1]. The changing landscape of schistosomiasis burdens brought about by such strategies [1], the altered geographical distribution of the parasites arising from changes to irrigation patterns and consequent effects on snail host distribution [2], and the looming spectre of resistance to the primary drug, praziquantel, emphasise the immediate need for highly specific and sensitive parasite surveillance methodology to be deployed [3].

Despite continuing improvements in immunological and molecular tools for schistosome diagnosis, a primary diagnostic technique, at least in field situations, remains direct observation of eggs in human faeces (*Schistosoma mansoni* and *S. japonicum*) and urine (*S. haematobium*). Choice of morphological tests, particularly the Kato-Katz method of diagnosis for intestinal forms of schistosomiasis, is driven by considerations of cost [4] and relative ease of use. The Kato-Katz method, however, although definitive because of the distinct morphology of the eggs of all schistosomes infecting humans, can be plagued by low sensitivity, especially in areas of low endemicity and particularly in individuals with low worm burdens.

## Paramagnetic Beads for Schistosome Diagnosis

A recent article by Fagundes Teixeira and colleagues [5] in this journal describes the intriguing discovery that paramagnetic beads, under the influence of a magnetic field, can be used to partially purify *S. mansoni* eggs from faeces for subsequent morphological identification. The original premise of the work was that eggs could be purified from faecal samples in solution by biotinylated lectins and streptavidin-conjugated beads. Lectins were theorised to bind carbohydrate moieties on eggshells and, using the strong affinity of streptavidin for biotin, egg-paramagnetic bead complexes could be entrapped in a magnetic field for subsequent purification. The authors were able to purify eggs, but not in the manner they had hypothesised, for it turned out that the beads themselves, not the lectin intermediates, were binding the schistosome eggs. With further exploration, they showed that antibody-conjugated beads could also bind eggs, although with less efficiency. Latex beads conjugated to protein-A did not allow for egg purification. From these results, the authors argued that the beads themselves, under the influence of an external magnetic field, could bind the eggs.

Streptavidin beads were more efficient than antibody-bound beads in binding eggs. This observation represents one limitation of the study, for the differential binding was not explored further to elucidate whether streptavidin itself plays a role in binding eggs or whether binding is dependent entirely upon magnetic interactions between the beads and the eggs. Nevertheless, the authors then used the beads to assess the diagnostic sensitivity of the technique. They found that sensitivity was 100% at egg burdens of 1.3 eggs per gram, dropping to 25% sensitivity at 0.1 eggs per gram. Clearly the technique, currently being patented under the name of Helmintex, is significantly more sensitive than the Kato-Katz method.

## Mechanisms for Egg Binding?

How can this affinity of the paramagnetic beads for schistosome eggs be explained? A possible answer comes from recent studies of iron (Fe) metabolism of schistosomes [6]. Earlier work had shown that female schistosomes accumulate iron in vitellocytes, cells of the female germinal line that synthesise eggshell precursors [7],[8]. The role of Fe in early development of schistosomes, however, remained uncertain. Energy dispersive spectroscopy of *S. japonicum* eggshells demonstrated that Fe is incorporated into the eggshell [6]. Schistosome eggshells are quinone-tanned, a chemical process in which eggshell precursors are modified under the action of tyrosinase enzymes [9]. Tyrosinases catalyse both the hydroxylation of tyrosine residues in eggshell precursor proteins to dihydroxy-phenylalanine and the subsequent oxidation to dopaquinone. In other invertebrates, the cross-linking reaction appears to be highly dependent on Fe, which acts to stabilise and strengthen the bridge (see [6]). The hypothesis, then, is that vitellocyte Fe is used by female worms in eggshell stabilisation. The explanation for the affinity of the paramagnetic beads and schistosome eggs is that it is the presence of Fe in eggshells that allows the affinity of the beads for eggshells in the presence of an external magnetic field. Under action of such a field, the paramagnetic beads themselves become magnetic, enabling them to interact with the eggs.

## Potential Outcomes

The paper by Fagundes Teixeira et al. [5] raises two interesting concepts for schistosomiasis studies. Firstly, the work reminds us that there are many fundamental questions to be answered with regard to the structural biology and chemistry of egg formation in schistosomes. Schistosome eggs have been largely refractory to structural analyses because they are resistant to hydrolysis by enzymes and because they appear to be synthesised from multiple precursor proteins [10]. Further studies of the structural biology of eggshells are of great importance because of the central role that eggs play in the schistosome–human host interplay and resultant morbidity.

Secondly, and of more immediate relevance, is the fact that the authors have uncovered a potential new tool for schistosome diagnosis that has greater sensitivity than the Kato-Katz method and is possibly less expensive than molecular and immunodiagnostic tools. As the authors point out, the method may be useful as an adjunct test in clinical settings, particularly for diagnosis of schistosomiasis in returned travellers. At US$0.80 per specimen [5], however, the Helmintex test is considerably more expensive than Kato-Katz thick smear examination, and it remains to be seen how well it can be applied in diverse field settings. The authors argue that an immediate application for the test would be seen in regions of low endemicity, where greater diagnostic precision could be obtained with Helmintex when compared to current methods.

Whether Helmintex will have an impact on schistosomiasis diagnosis, however, will depend on its relative cost-effectiveness. Kato-Katz examination, despite its low sensitivity, remains a competitive and cost-effective screening technique for intestinal schistosomiasis in regions with a range of prevalences [4]. The cost-effectiveness of Helmintex will need to be weighed against that of the Kato-Katz technique to determine whether it has wide applicability in a variety of field settings. In addition, trends towards integrated examination and control of schistosomiasis, soil-transmitted helminthiasis, and other neglected tropical diseases [11] may favour retention of simpler diagnostic tools, particularly if nematode eggs do not co-migrate with paramagnetic beads in the manner described for schistosome eggs [5].
